# Branched Chain Amino Acid Suppresses Hepatocellular Cancer Stem Cells through the Activation of Mammalian Target of Rapamycin

**DOI:** 10.1371/journal.pone.0082346

**Published:** 2013-11-27

**Authors:** Shinobu Nishitani, Mayumi Horie, Sonoko Ishizaki, Hirohisa Yano

**Affiliations:** 1 Exploratory Research Laboratories, Research Center, Ajinomoto Pharmaceuticals, Co, Ltd, Kanagawa, Japan; 2 Department of Pathology, Kurume University School of Medicine, Kurume, Japan; Institut für Pathologie, Greifswald, Germany, Germany

## Abstract

Differentiation of cancer stem cells (CSCs) into cancer cells causes increased sensitivity to chemotherapeutic agents. Although inhibition of mammalian target of rapamycin (mTOR) leads to CSC survival, the effect of branched chain amino acids (BCAAs), an mTOR complex 1 (mTORC1) activator remains unknown. In this study, we examined the effects of BCAA on hepatocellular carcinoma (HCC) cells expressing a hepatic CSC marker, EpCAM. We examined the effects of BCAA and/or 5-fluorouracil (FU) on expression of EpCAM and other CSC-related markers, as well as cell proliferation in HCC cells and in a xenograft mouse model. We also characterized CSC-related and mTOR signal-related molecule expression and tumorigenicity in HCC cells with knockdown of Rictor or Raptor, or overexpression of constitutively active rheb (caRheb). mTOR signal-related molecule expression was also examined in BCAA-treated HCC cells. *In-vitro* BCAA reduced the frequency of EpCAM-positive cells and improved sensitivity to the anti-proliferative effect of 5-FU. Combined 5-FU and BCAA provided better antitumor efficacy than 5-FU alone in the xenograft model. Stimulation with high doses of BCAA activated mTORC1. Knockdown and overexpression experiments revealed that inhibition of mTOR complex 2 (mTORC2) or activation of mTORC1 led to decreased EpCAM expression and little or no tumorigenicity. BCAA may enhance the sensitivity to chemotherapy by reducing the population of cscs via the mTOR pathway. This result suggests the utility of BCAA in liver cancer therapy.

## Introduction

The term “cancer stem cell” (CSC) refers to a cancer cell with the characteristics of a stem cell. Stem cells carry the potential to self-renew and differentiate into other cell types, and can therefore restore cells undergoing apoptosis. It is hypothesized that carcinomas developing from stem cells undergo a process of asymmetric division. CSCs were initially identified in acute myeloid leukemia [1] and have subsequently been discovered in other cancers. A challenge arose when it was thought that CSCs were resistant to therapy. Many chemotherapeutic agents target active proliferating cells and may not be effective against cells undergoing limited proliferation. Surviving cancer cells were determined to be a factor of recrudescence or metastasis to other tissues [2,3]. Studies have found many CSC markers in various cancers. In hepatocellular carcinoma (HCC), CD133 and EpCAM have been identified as CSC markers [4]. Furthermore, CSCs express ABC transporters when actively exposed to chemotherapeutic agents, contributing to therapeutic resistance [5]. A combination study evaluating these treatment-refractory CSCs found that existing cancer cells could be completely rooted out [6].

Two approaches to cancer therapy have been suggested: Wake up therapy and Sleep therapy. Wake up therapy enhances sensitivity to chemotherapeutic agents by inducing differentiation from CSCs to cancer cells. Oncostatin M, a cytokine of IL-6 family, is a well-known inducer of differentiation. Combination of this cytokine with a chemotherapeutic agent improved antitumor efficacy in an HCC xenograft model [7]. However, oncostatin M has not been clinically tested. Sleep therapy reportedly maintains low proliferation of CSCs, but the pathophysiology of this phenomenon remains unclear. Studies have mainly focused on hematopoietic cells with mammalian target of rapamycin (mTOR) signals associated with differentiation [8,9]. It is well known that branched chain amino acids (BCAAs), especially leucine, activate mTORC1 [10,11]. BCAAs are necessary for ammonium metabolism in muscles when the liver is unable to perform this function. Recent reports have shown that BCAA activates albumin synthesis in rat primary hepatocytes [12] and cirrhotic rat liver [13] through mTOR signaling, a central regulator of protein synthesis, by detecting nutritional conditions [14]. In Japan, pharmacological supplementation with BCAA granules is used to treat hypoalbuminemia in patients with decompensated liver cirrhosis (LC) [15]. BCAA granules, prescribed three times a day after meals to provide a total daily dose of 12 g of BCAA, have a 2:1:1.2 weight ratio of leucine to isoleucine to valine. BCAA suppresses the occurrence of HCC in animal models [16,17] and LC patients [18]. We focused on inducing CSC differentiation, particularly the enhancement of chemotherapeutic sensitivity. CSC differentiation was evaluated by activating mTORC1 with BCAA, and the anti-tumor effect of the combination of BCAA and chemotherapeutic agent was studied *in vitro* and *vivo*. Our results will be of considerable value to understanding the clinical efficacy of liver carcinoma therapy in patients with LC.

## Materials and Methods

All animal work was conducted according to relevant national and international guidelines. 

### Cell culture and reagents

Two HCC cell lines, HAK1-B [19] and Huh7, were maintained in RPMI1640 (Nacalai Tesque, Japan) and DMEM (Nacalai Tesque, Japan) respectively or LC medium, which is a medium with low Fischer ratio (ratio of BCAA concentration over aromatic acid concentration) [20] containing 1% penicillin/streptomycin and 10% fetal bovine serum (FBS). Reagents used included anti-EpCAM FITC conjugated antibody (Abcam, USA), Hoechst 33342 solution (DOJINDO, Tokyo, Japan) for nuclear staining and cell number counting by array scan system (Thermo Fischer technology, USA); 5-Fluorouracil (5-FU) was obtained from Kyowa Kirin, Japan.

### Plasmid construction of Rheb constitutive active form

Flag-caRheb plasmid was provided by Dr. Tomohiko Maehama (The Tokyo Metropolitan Institute of Medical Science, Tokyo, Japan).

### shRNA plasmids

Small hairpin RNA (shRNA) plasmids were obtained from iGENE therapeutics (USA). 

The shRNA lipofectamine system was designed according to the manufacturer’s instructions. The target sequences of shRNA were as follows: sh-Raptor: 5′-GCGGAAAGGATTATGGGT-3′; sh-Rictor: 5′-GTAGAAAGTTCAACGAGCT-3′; and U6RepT7STOP (sh-RNA control vector): 5′-CACCTTTTTTTAAAAAAATCCT-3′.

### Quantitative real-time polymerase chain reaction (Q-PCR)

Q-PCR was used to detect mRNA levels of CYP3A4, Bmi1, EpCAM, FOXO3a, Raptor, and Rictor. A total of 1 × 10^5^ HAK-1B cells were seeded in RPMI1640 medium containing 10% FBS for 24 h prior to experiments. BCAA (4 mM) was added to the medium and maintained for 72 h. RNA was isolated using TriPure isolation reagent (Roche Applied Science) and complementary DNA (cDNA) was synthesized using the High Capacity cDNA reverse transcription kit (Applied Biosystems, Carlsbad, CA). Real-time polymerase chain reaction (PCR) was performed using the 7500 Real-Time PCR System (Applied Biosystems) and Power SYBR Green PCR Master Mix (Applied Biosystems) containing specific primers, according to the manufacturer’s instructions. Each sample was normalized to GAPDH expression.

Primer sequences for PCR were as follows: CYP3A4: forward 5′-TTGGAAGTGGACCCAGAAAC-3′, reverse 5′-CTGGTGTTCTCAGGCACAGA-3′; Bmi1: forward 5′-CCAGGGCTTTCAAAAATGA-3′, reverse 5′-GCATCACAGTCATTGCTGCT-3′; EpCAM: forward 5′-GCTGGTGTGTGAACACTGCT-3′, reverse 5′-ACGCGTTGTGATCTCCTTCT-3′; 

FOXO3a: forward 5′-TGCTGTATGCAAGAACTTTCCAGTAGCAG-3′, reverse 5′-ACTCTAGCCCCCATGCTACTAGTG-3′; Raptor: forward

5′-CCCTGCTACTCGCTTT-3′, reverse 5′-GTGAGGTGTTTCCCCT-3′;

Rictor: forward 5′-GGAAGCCTGTTGATGG-3′, reverse 5′-GGCAGCCTGTTTTATG-3′

### Cell count and EpCAM-positive cell detection assay

Cells were cultured in the presence or absence of BCAA for 72 h, then fixed using 4% paraformaldehyde and stained with Hoechst 33342 (10 mg/mL, Dojindo, Tokyo, Japan) for 1 min. Cell counts were performed using the target activation protocol and an array scan system. EpCAM-positive cells were detected by staining the fixed cells with EpCAM-conjugated FITC antibody for 1 h, and the number of EpCAM-positive cells per 5000 cells were detected by the scan system 

### Apoptosis assay

Cells were cultured with 5-FU (0, 1, 2 μg/mL) in the presence or absence of 2 mM BCAA for 72 h in 96-well plates (BD Biosciences). Annexin V binding to cell membranes was visualized with an Annexin V-FITC detection kit (TAKARA BIO INC, Tokyo, Japan) and Hoechst 33342 solution by array scan.

### Western blotting

A total of 1 × 10^5^ Huh7 cells were seeded in DMEM medium 24 h prior to study experiments. The medium was exchanged with LC medium for 3 days or DMEM medium with various treatments: knockdown for 5 days, overexpression for 1 day, and BCAA treatment for 30 min or 3 days. Western blotting was performed in the usual manner. Cells were washed in phosphate-buffered saline (PBS) and lysed in RIPA buffer containing complete protease and phosphatase inhibitor cocktail (Roche Applied Science, Indianapolis, INC). The membranes were blocked in Blocking One-P (Nacalai Tesque, Japan). Antibodies included rabbit anti-Rictor (Cell Signaling Technology, Beverly, MA), rabbit anti-raptor (Bethyl Laboratories, Montgomery, TX), rabbit anti-p-70S6 kinase, anti-total-p-70S6 kinase, rabbit anti-p-4EBP1 (T37/46), rabbit anti-p-Akt (T308 or S473), rabbit anti-p-GSK3β (S9), rabbit anti-β-catenin (Cell Signaling Technology, Beverly, MA), and mouse anti-α-tubulin (Sigma-Aldrich, St Louis, MO). Densitometry was conducted directly on the blotted membrane using a charge-coupled device camera system (LAS-4000 Mini, Fujifilm, Tokyo, Japan). 

### Knockdown Experiments

Huh-7 cells were transfected with negative control (NC) or target (mTORC1 or mTOR2 regulatory associated protein of Raptor and Rictor) small hairpin RNA (shRNA) using Lipofectamine™ LTX Reagent (Invitrogen Technology, USA) according to the manufacturer’s instructions. Stable transfectants were selected, as previously described [21], for resistance to puromycin for 2 days and subsequently cultured in DMEM containing 10% FBS for 2 days. Q-PCR and western blotting confirmed knockdown efficacy.

### Animal studies

#### Animals

The following experimental protocol was reviewed and approved by the Animal Care Committee of Ajinomoto Co., Inc. Female BALB/c nude mice or NOD/SCID mice at age 6 weeks were obtained from Charles River Japan (Yokohama, Japan). They were maintained in individual cages in a clean, air-conditioned room (24 ± 1°C) with a 12 h-12 h light-dark cycle (lights on from 0700 to 1900). The animals were fed a stock sterile powder diet (CRF-1, Oriental Yeast, Tokyo, Japan).

#### Chemotherapy

One million HAK-1B cells were suspended in 100 μL of RPMI1640 with FBS, and a subcutaneous injection was performed. The incidence and size of subcutaneous tumors were recorded when the average volume had reached 100 mm^3^ as previously described [7]. Tumor injections of 50 μL of 10% DMSO/PBS (control), or 5-FU (250 μg/tumor) were given twice weekly and 3% BCAA-supplemented or 3% casein-supplemented diet was provided daily for 2 weeks starting 7 days after tumor cell injection. Tumor volumes were evaluated using caliper measurements and calculated with the formula V= 1/2 × *a* ^2^ × *b* where *a* is the short axis and *b* is the long axis of the tumor. On the 14^th^ day, all mice were sacrificed under anesthesia, isolated tumors were weighed, their volumes calculated, and tissue analyzed for mRNA expression.

#### Tumorigenesis

One million Huh7 cells transfected with lipofectal particles containing NC, Raptor, or Rictor shRNA, control plasmid cDNA (pcDNA), or Flag caRheb plasmid were harvested and injected subcutaneously into NOD/SCID mice (n = 5 in each group). Tumor volumes were evaluated as before. Mice bearing the NC, knockdown (KD), or over expression xenografts were sacrificed after 4 weeks. 

#### Statistical methods

Results were expressed as mean ± SE. Significance was determined in EXSUS version 7.7.1 (CAC Corporation, Tokyo, Japan) by performing Dunnett’s test, Tukey’s test and Student’s *t*-test, and differences were considered significant at P values < 0.05.

## Results

### The effect of BCAA on EpCAM-positivity in HAK-1B cells

**Figure 1:**
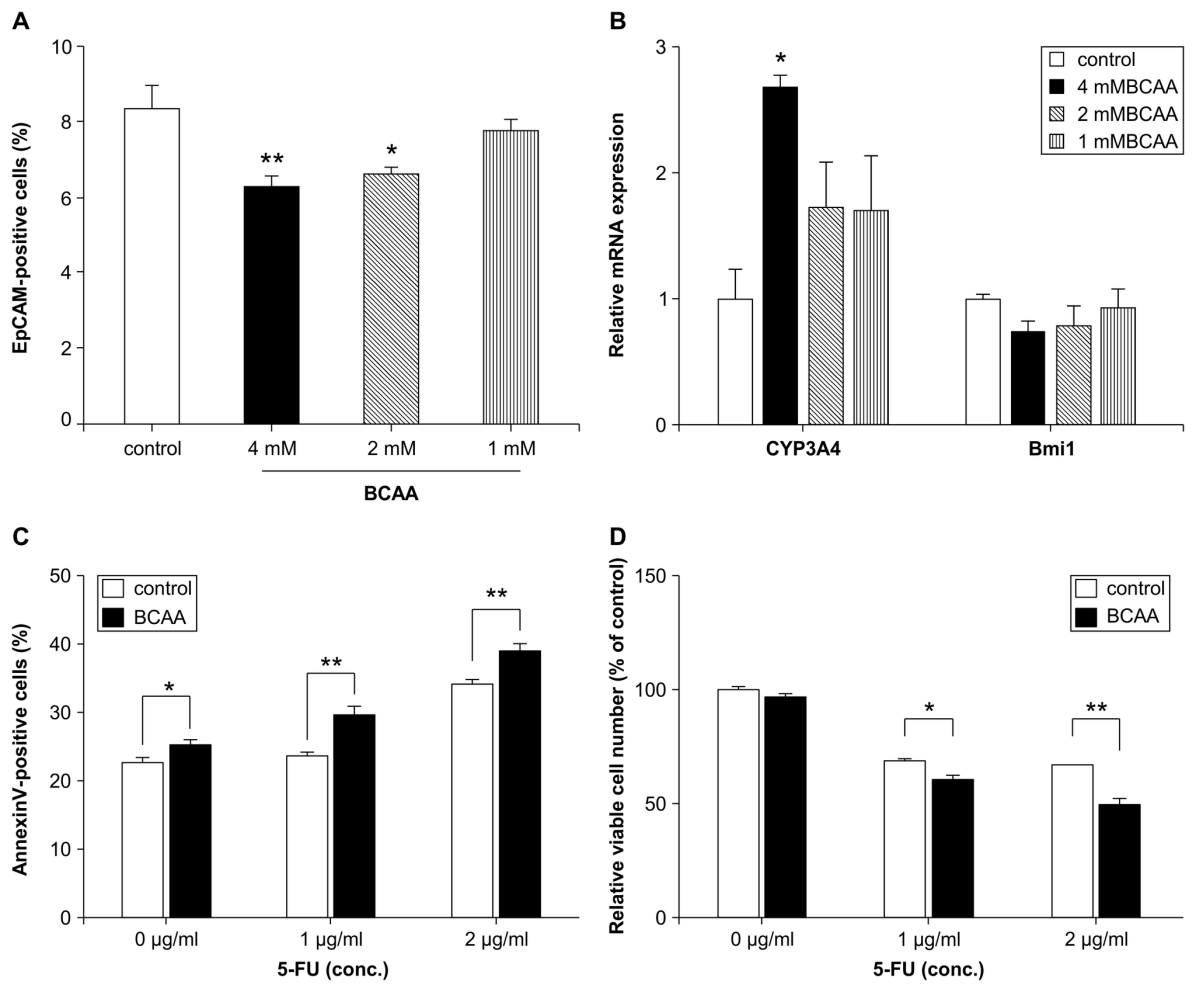
Changes in the percentage of EpCAM-positive cells by using the target activation array scan protocol (A), and CYP3A4 and Bmi mRNA levels (B) in HAK1-B cells cultured in RPMI1640 containing 10% FBS only, or with 1, 2, or 4 mM BCAA added for 72 h. Dunnett's test, *p < 0.05, **p < 0.01 n = 6, mean ± SE. The percentage of Annexin V-positive cells (C) and relative viable cell number (D) by array scan in HAK-1B cells cultured in RPMI1640 containing 10% FBS with or without 2 mM BCAA in the presence (1 or 2 µg/mL) or absence of 5-FU for 72 h by using target activation protocol of array scan. Student *t*-test, *p < 0.05, **p < 0.01, n = 7, mean ± SE.

HAK-1B cells were approximately 10% EpCAM-positive (Figure S1); this decreased significantly in a dose-dependent manner in the presence of 1–4 mM BCAA (Figure 1A). 4 mM BCAA led to an increase in the expression CYP3A4 mRNA, a differentiated marker, and by contrast 1-4mM BCAA tended to decrease in the expression of Bmi1 mRNA, an undifferentiated marker (Figure 1B). These results suggest BCAA reduced EpCAM-positivity via differentiation. Hence, we evaluated chemotherapeutic sensitivity of HAK-1B containing EpCAM-positive cells. Annexin V expression, which was used to detect the primary apoptosis event, increased with increasing doses of 5-FU; this increase was greater in cells treated with combination BCAA and 5-FU (Figure 1C). In addition, HAK-1B proliferation was more strongly inhibited by a combination of BCAA and 5-FU than by 5-FU alone (Figure 1D). 

The same results were obtained with Huh7 cells and are showed in Figure S2 A-B.

### Chemotherapeutic sensitivity *in vivo*

To test the hypothesis that EpCAM positivity increases chemotherapeutic sensitivity in the presence of BCAA, we used combined BCAA and 5-FU treatment in a BALB/c nude mouse model transplanted with subcutaneous HAK-1B. 

**Figure 2:**
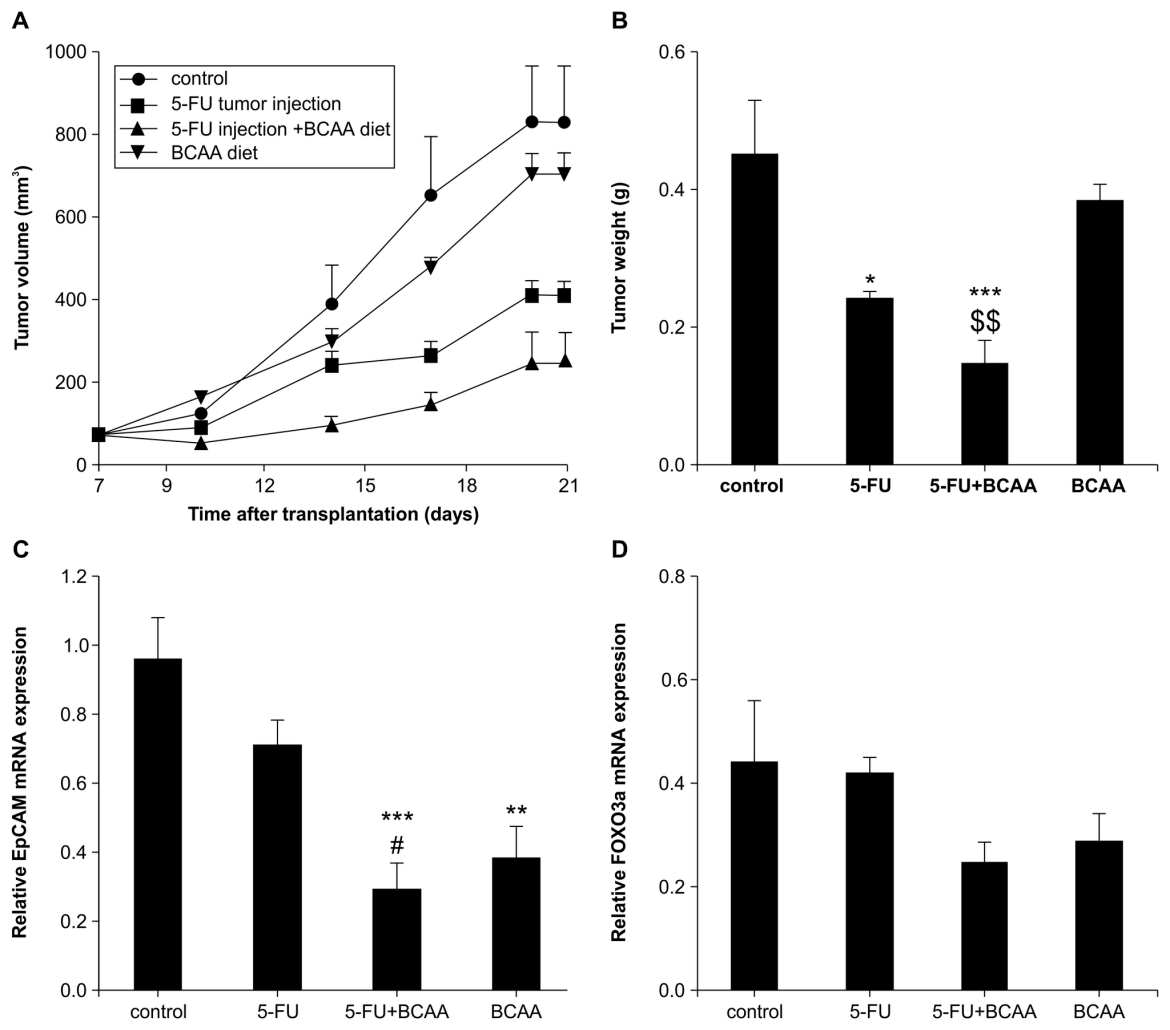
Tumor volume change over 14 days (A) and tumor weight (B) in the HAK-1B xenograft mouse model on the 14^th^ day after administration of BCAA and 5-FU injection. The relative expression of mRNA of various molecules of each tumor was associated with CSC properties (C, D). Control: 10% DMSO/saline/tumor injection + 3% casein containing diet, 5-FU: 250 µg/tumor injection + 3% casein containing diet, BCAA diet: 10% DMSO/saline/tumor injection + 3% BCAA containing, 5-FU+BCAA diet: 250 µg/tumor injection + 3% BCAA containing diet for 14 days. Tukey’s test: *p < 0.05, **p < 0.01, ***p < 0.001 vs. control, #p < 0.05 vs. 5-FU, $$p < 0.01 vs. BCAA, n = 6, mean ± SE.

Antitumor efficacy was significant with 5-FU alone, and an even stronger antitumor effect was achieved with combination BCAA and 5-FU treatment. However, the antitumor effect of BCAA alone was not remarkable (Figure 2A, B, Figure S3). EpCAM mRNA expression was significantly decreased in BCAA-treated mouse tumors (Figure 2C). FOXO mRNA (Figure 2D), reported to be expressed in CSCs [22], was significantly reduced in a similar manner. 

### Mechanism of BCAA effects on EpCAM-positive Huh7 cells

**Figure 3:**
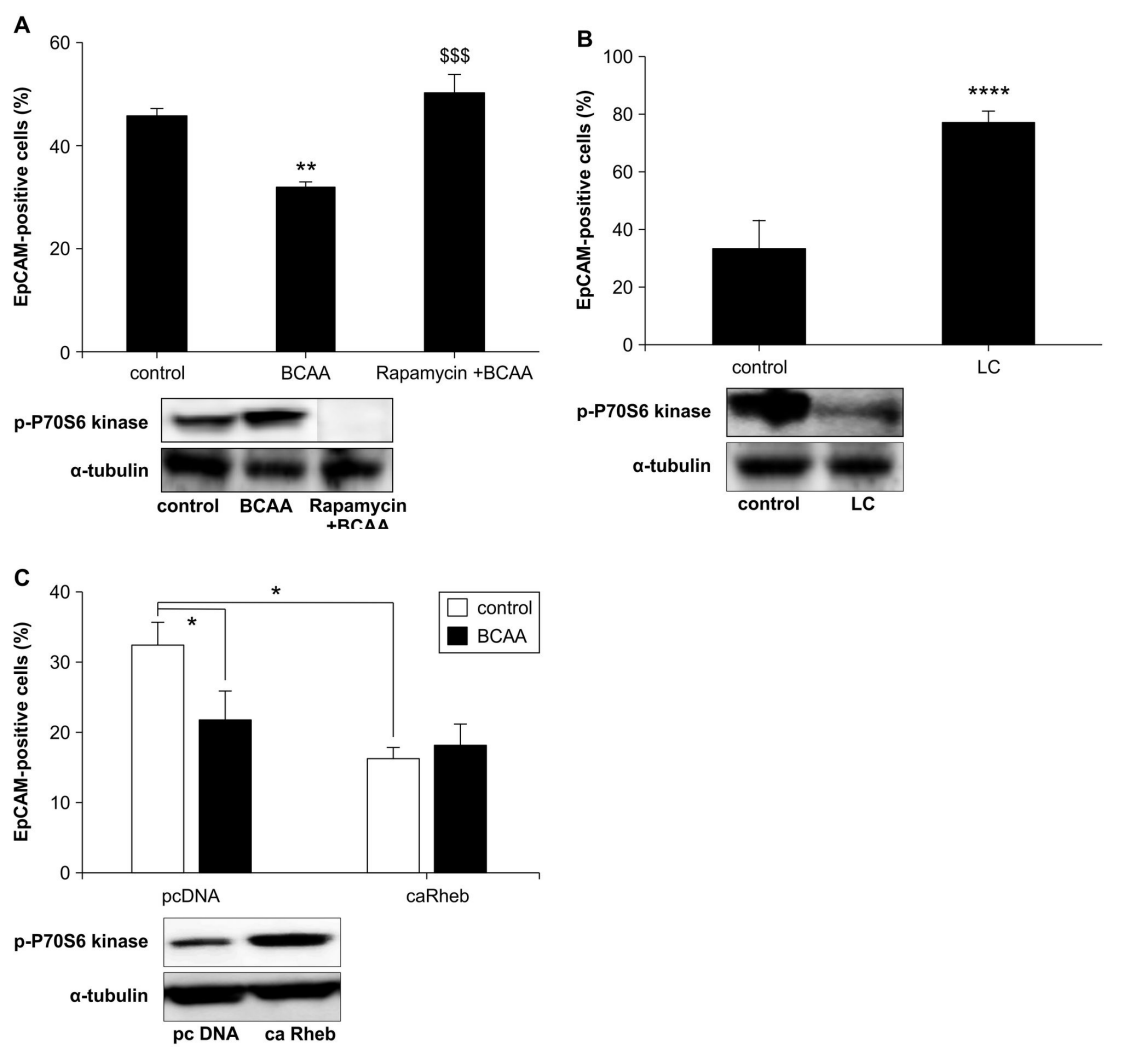
Changes in the percentage of EpCAM-positive cells upon control medium (DMEM containing 10% FBS) with 4 mM BCAA stimulation or 100 nM rapamycin pretreatment and 4 mM BCAA stimulation (A) or liver cirrhosis modified medium (LC) containing 10% FBS stimulation (B) for 72 h in Huh7 by using the target activation protocol of array scan. The rate change of EpCAM-positive cells in 5000 cells with overexpression of caRheb or control plasmid cDNA (pc DNA) in control medium (DMEM containing 10% FBS) with and without 4 mM BCAA stimulation for 24 h in Huh7 (C). The detection of P70S6 kinase phosphorylation, a member of downstream mTOR signaling, in the presence of DMEM, BCAA treatment, pretreatment with rapamycin and BCAA treatment, or LC stimulation for 72 h in Huh7 (A,B). Tukey’s test **p < 0.01 vs. control, $$$p < 0.001 vs. BCAA, n = 8, mean ± SE (A). Student t-test *p < 0.05, ****p < 0.0001, n = 8, mean ± SE (B,C).

Considering the experimental transfection efficiency of Huh7 cells, we used them to evaluate the mechanism of BCAA, assuming that 40% of Huh7 were EpCAM-positive as previously reported [7]. As in HAK-1B, EpCAM-positive cells in Huh7 were significantly reduced by BCAA; in addition, mTORC1 was activated upon BCAA treatment and inhibited by rapamycin (Figure 3A, Figure S4A). The decrease in EpCAM-positive cells due to BCAA was completely abrogated by pretreatment with rapamycin, an inhibitor of mTORC1 (Figure 3A, Figure S4A). In addition, LC medium, a low Fischer ratio medium, inhibited mTORC1 activity and increased the rate of CSCs (Figure 3B). mTORC1 activation was suppressed by LC medium (Figure 3B, Figure S4B). 

Finally, EpCAM-positive cells were significantly reduced by mTORC1 activation via overexpression of caRheb, an activating factor of mTORC1 without BCAA stimulation (Figure 3C). Thus, the effect of BCAA on EpCAM-positive cells depended on mTORC1 activation. 

### The association between mTOR signal and EpCAM-positivity

The mTOR signals include two subtypes: mTORC1 and mTORC2 [23]. In particular, mTORC1 has Raptor, a regulatory protein of the mTORC1 complex, and is stimulated by BCAA, especially leucine. In contrast, mTORC2 has Rictor, a regulatory protein of mTORC2 complex, which signals Akt downstream. 

**Figure 4:**
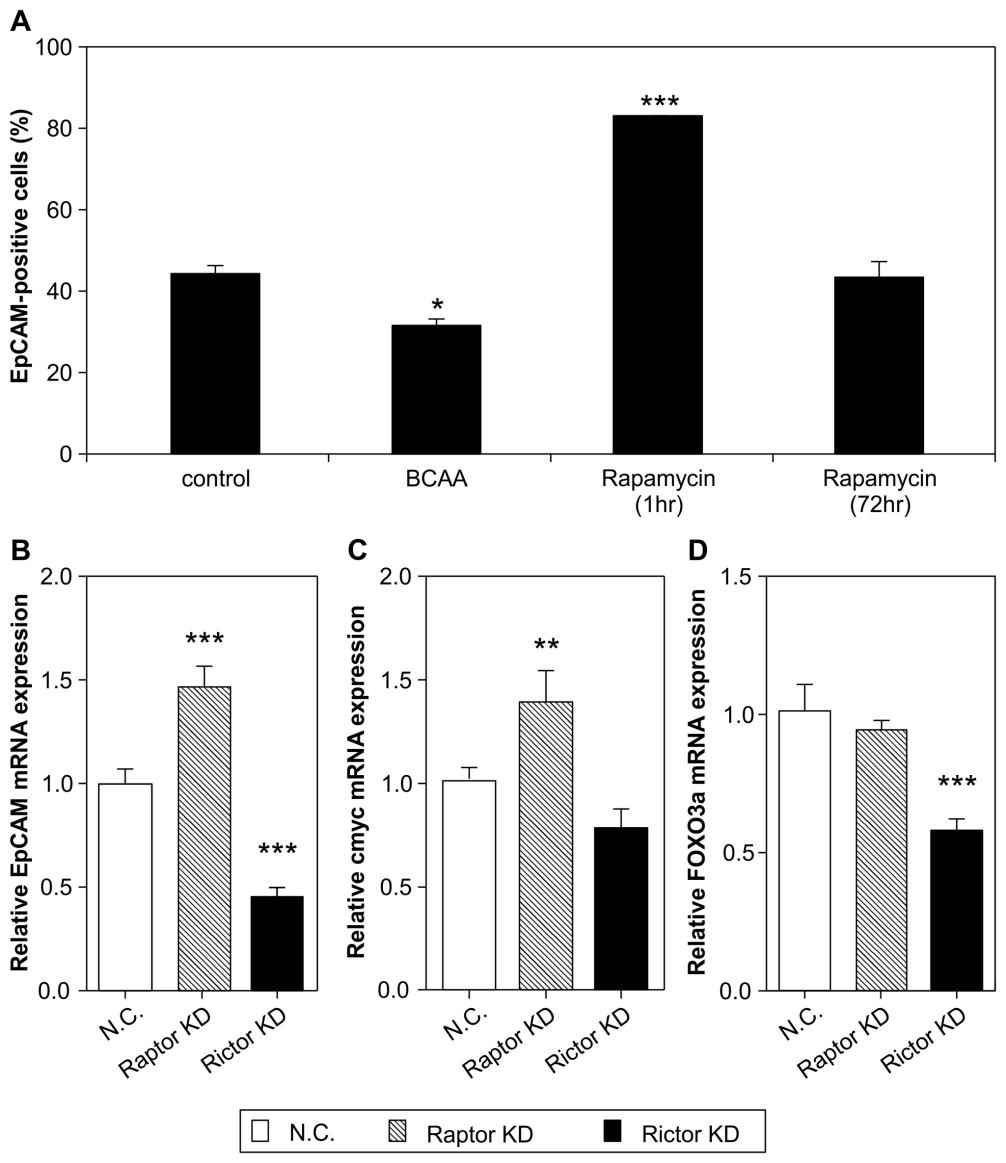
The rate change of EpCAM positive cell in 5000 cells in the presence of 100 nM rapamycin treatment for 1 h or 72 h (A). Dunnett's test, *p < 0.05, ***p <0.001 vs. control, n = 6, mean ± SE. The relative expressions of EpCAM, c-myc, and FOXO3a mRNA upon Raptor and Rictor knockdown (B-D). Dunnett's test, **p < 0.01, ***p < 0.001 vs. control n = 8, mean ± SE.

Rapamycin inhibits mTORC1 and mTORC2, depending on the duration of stimulation [24,25]. Thus, we can determine the effects of inhibiting mTORC1 alone or dual inhibition of mTORC1/2 on EpCAM-positive cells by pretreatment with rapamycin for 1 h or 24 h, respectively. We found that EpCAM-positive cells increased significantly with inhibition of mTORC1. In contrast, EpCAM-positive cells decreased with dual inhibition of mTORC1/2, especially mTORC2 inhibition, by pretreatment for 72 h (Figure 4A). 

These findings suggest the presence or absence of EpCAM-positive cells depends on mTORC1 or mTORC2, respectively. We then examined the expression of EpCAM mRNA in the presence of mTORC1 or mTORC2 loss of function by knockdown of Raptor or Rictor, respectively. Loss of mTORC1 function caused EpCAM mRNA expression to increase similarly to mTORC1 inhibition by pretreatment with rapamycin for 1 h. However, loss of mTORC2 function caused EpCAM mRNA expression to decrease significantly. Furthermore, c-myc and FOXO3a mRNA expression reacted similarly to EpCAM mRNA expression (Figure 4B-D). 

**Figure 5:**
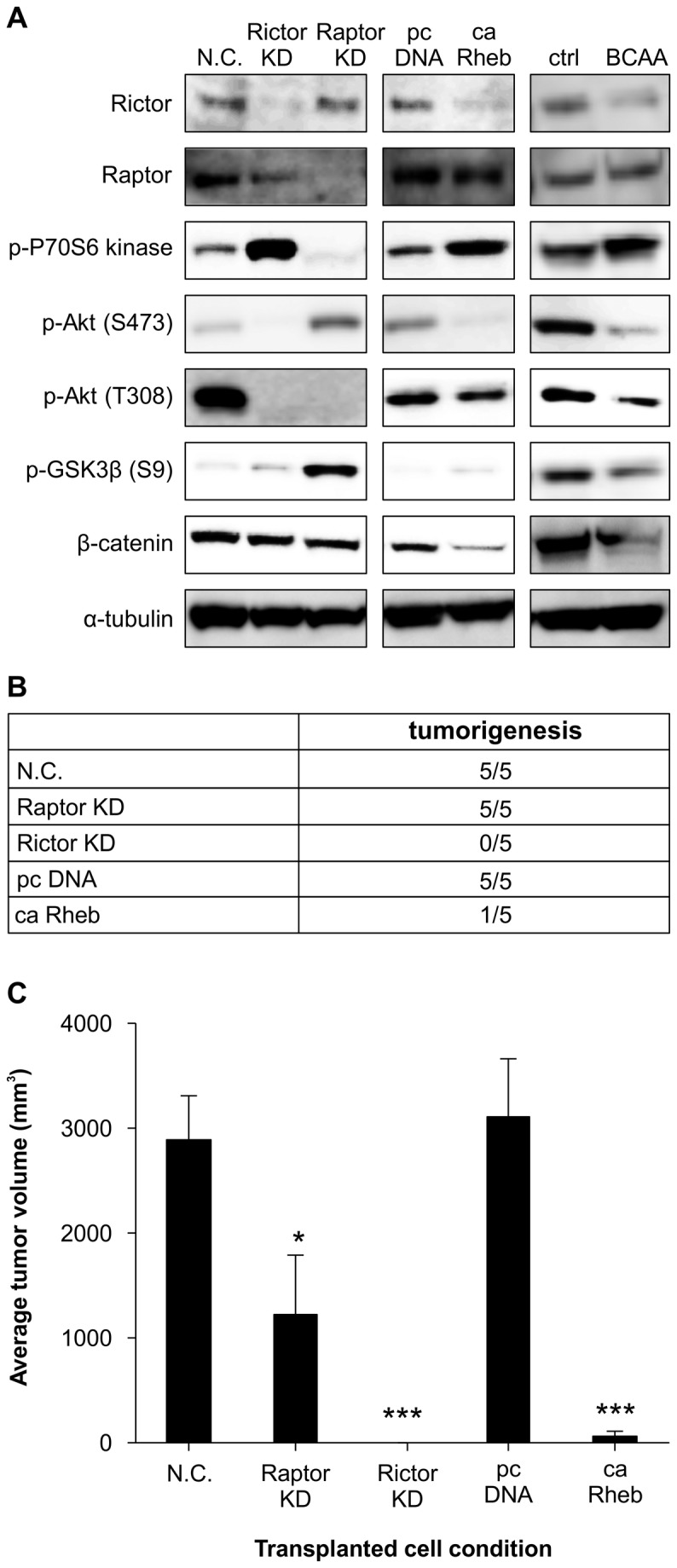
Protein expression and phosphorylation in Huh7 cells under Knockdown conditions for 5 days and overexpression conditions for 1 day, and detection of phosphorylation after 4 mM BCAA treatment for 30 min. Rictor or Raptor Knockdown compared to negative control (NC), caRheb compared to control plasmid cDNA (pc DNA), BCAA treatment compared to DMEM (FBS 10%) only (Ctrl) (A). The average tumor volumes and tumorigenesis ratio at the 4^th^ week in a xenograft model with transplanted cells with negative control, knockdown of Raptor, Rictor for 5 days, or overexpression of control plasmid DNA, caRheb for 1 day (C), and tumorigenesis rate (B). Dunnett's test, *p<0.05, ***p < 0.001 vs. N.C. n = 5, mean ± SE.

Rictor, Raptor, and β-catenin were also associated with mTOR and Wnt/β-catenin signals (Figure 5A). When mTORC1 was inhibited by Raptor knockdown, phosphorylation of p70S6 kinase, the downstream signal of mTORC1, decreased, and phosphorylation of Akt (Ser473) increased. This suggests that the inhibition of mTORC2 phosphorylation by P70S6kinase was abrogated by Raptor knockdown. In contrast, when mTORC2 was inhibited by Rictor knockdown, phosphorylation of p70S6 kinase increased, and phosphorylation of Akt decreased. 

When mTORC1 was activated by caRheb overexpression or 4 mM BCAA treatment, the phosphorylation of p70S6 kinase increased and phosphorylation of Akt decreased. Furthermore, β-catenin protein was decreased by inhibition of phosphorylation of GSK3β via mTORC1 activation. 

In addition, GSK3β was phosphorylated by Akt (Ser473) in response to Raptor knockdown; however, phosphorylation of GSK3β and β-catenin protein was not affected by Rictor knockdown. 

### Tumorigenesis of mTOR-knockdown and mTORC1-activated cells

The activation of mTORC1 or inhibition of mTORC2 was hypothesized to repress EpCAM-positive cells. The tumorigenic ability of CSCs was examined using a xenograft model subcutaneously implanted with Raptor or Rictor knockdown, control plasmid cDNA, or caRheb overexpressing Huh7 cells in mice for 4 weeks (Figure 5B and 5C). 

BCAA activated mTORC1; however, BCAA has dual effects on mTORC1. Stimulation with high doses of BCAA decreased EpCAM-positive cells via activation of mTORC1, while stimulation with low doses increased EpCAM-positive cells via inhibition of mTORC1 (Figure 3A and 3B). High-dose BCAA could not be maintained through tumor implantation, and tumorigenesis was not established when cells treated with high-dose BCAA were subcutaneously implanted. Instead, we used caRheb-overexpressing cells as mTORC1-activating cells for the tumorigenesis study, which could maintain mTORC1 activation for at least one week.

Although tumor size was smaller in the group implanted with Raptor knockdown cells than in the negative control group (Figure 5C), tumorigenesis was maintained in all 5 mice, similar to the negative control transfection cell implanted group (Figure 5B). However, the two groups consisting of Rictor knockdown cells and caRheb overexpressing cells had 0/5 mice or only 1/5 mice retain tumorigenesis potential, respectively. Furthermore, tumor size in the caRheb overexpression group was smaller than in the control plasmid cDNA group. 

## Discussion

One of the novel contributions of this study was the finding that cancer cells experienced increased sensitivity to chemotherapy agents after EpCAM-positive cells differentiated by BCAA treatment via mTORC1 activation (Figure 1). Additionally, low BCAA stimulation with LC medium with a low Fischer ratio (ratio of BCAA concentration over aromatic acid concentration), led to suppressed mTORC1 activation, resulting in increased EpCAM-positive cells (Figure 3B). In patients with chronic hepatitis or cirrhosis, hypoalbuminemia and decreased plasma values for Fischer ratio of BCAA to aromatic acids are commonly observed [26]. These patients may develop HCC or have a relapse of HCC. BCAA supplementation is known to improve the Fischer ratio in liver cirrhosis [27]. Hence, a higher Fischer ratio may prevent liver carcinogenesis or relapse due to CSCs. In Japan, BCAA granules are indicated for decompensated cirrhosis in patients with hypoalbuminemia despite adequate dietary intake, and oral administration of BCAA granules elevates the concentrations of plasma BCAA at about 1 mM [20]. After oral administration of BCAA in rats, the plasma concentration of BCAA in portal vein was several times higher than the concentration of BCAA in peripheral blood (data not shown). Therefore, we considered that the concentration of BCAA used *in vitro* (2^~^4 mM) was not significantly different from the concentration of BCAA *in vivo*. 

We also found that mTORC1 activation by BCAA treatment suppressed EpCAM-positive cells and enhanced the sensitivity of chemotherapeutic agents in HCC tumor (Figure 2). We examined EpCAM mRNA in liver after administration of BCAA in normal mice to investigate if there is any effect on the liver. As a result, BCAA did not show any effect on the expression of EpCAM in the liver (data not shown).

The mechanism of the effects of BCAA treatment on EpCAM-positive cells was evaluated by accelerated differentiation to cancer cells via activation of mTORC1. Phosphorylation of GSK3β was inhibited by activating mTORC1 and the downstream Wnt/β-catenin signal, while phosphorylation of β-catenin was maintained, resulting in β-catenin degradation. It was inferred that this inhibited the nuclear translocation of β-catenin, and subsequently inhibited transcription of, c-myc, EpCAM, and Bmi1. Furthermore, the downstream signals of EpCAM were determined to have a c-myc transcription signal [28], which correlated with the expression of EpCAM and c-myc. These suggested that EpCAM, c-myc expression decreased and mTORC1 activation increased the sensitivity to chemotherapy (Figure 2). 

Moreover, we found that mTORC1 activation decreased the protein expression of Rictor via overexpression of caRheb (Figure 5A). Studies have previously reported that activation of p70S6 kinase feedback inhibited Rictor phosphorylation, resulting in the suppression of Rictor function [29]. However, our data showed that protein expression of Rictor decreased. The mechanism by which mTORC1 activation decreased protein expression of Rictor remains unclear and is a potential future research direction. 

The inhibition of mTORC2 function by Rictor knockdown led to the activation of p70S6 kinase, a signal of mTORC1, decreased EpCAM, c-myc, and FOXO3a expression, and phosphorylation of Akt, but had no effect on GSK3β and protein expression of β-catenin. It was not clear whether p70S6 kinase was directly activated by Rictor knockdown, although it is possible that mTORC1 activation and inhibition of Akt signals led to decreased EpCAM expression. Several studies have suggested that inhibition of mTORC2, and not mTORC1, suppresses carcinogenesis [19,30]. In other reports, phosphorylation of Akt (Ser473) correlated significantly with cancer stemness [31,32]. Clinical research has reported that patients with high Rictor or EpCAM mRNA expression in isolated liver cancer had a higher rate of relapse [33,34]. 

**Figure 6:**
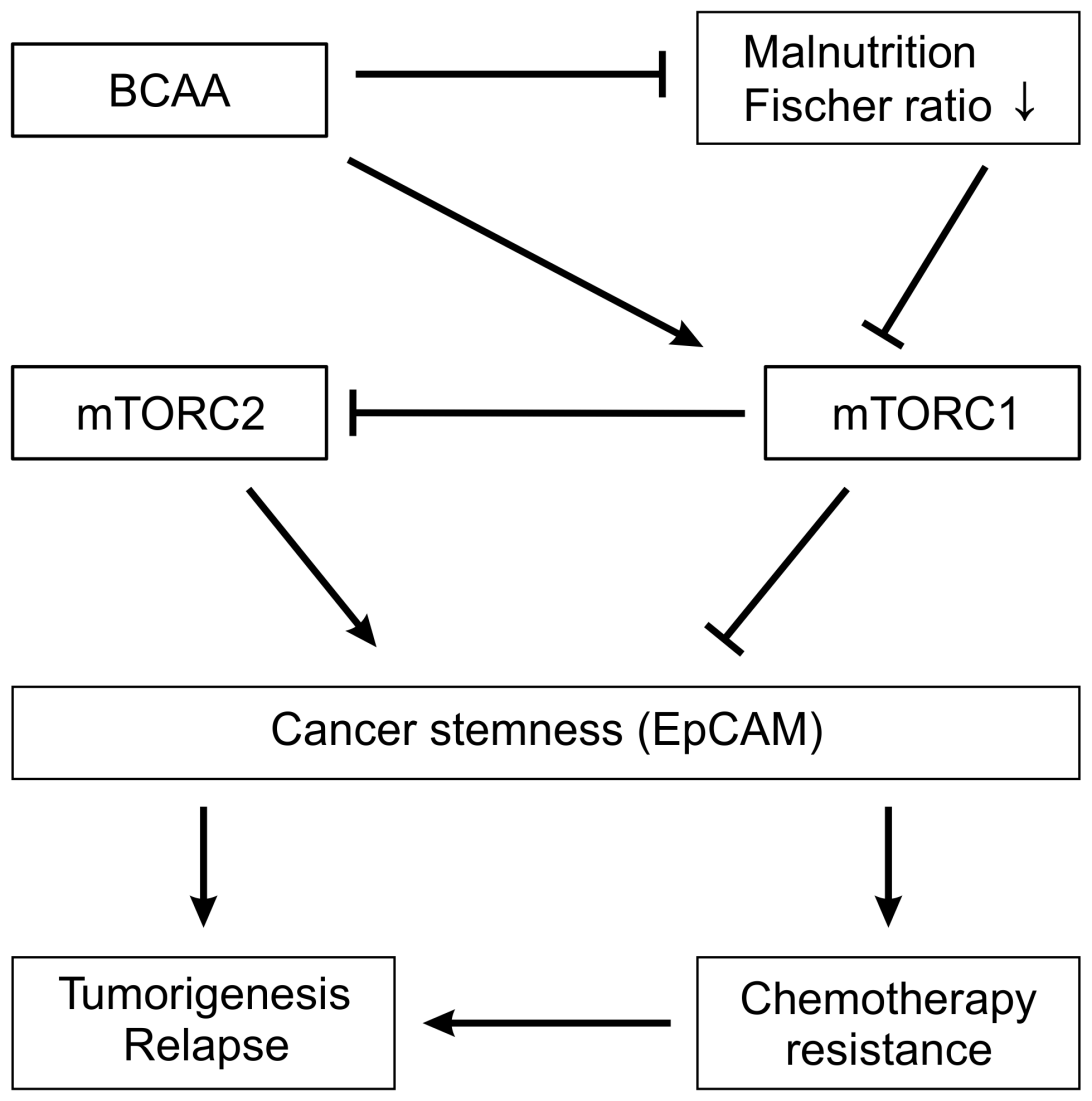
We found several new effects of BCAA and mTOR signaling on HCC cells in this study. Malnutrition or low Fischer ratio suppressed mTORC1 activation which led to increased cancer stemness. By contrast, mTORC2 maintains cancer stemness. However, mTORC1 inhibits mTORC2. BCAA suppresses cancer stemness by activation of mTORC1, and enhances antitumor effects of chemotherapy agents.

In this study, our findings suggest that CSC potential is strongly associated with mTORC signals. Furthermore, mTORC2 has a role in maintaining the stem cell potential whereas mTORC1 suppresses this potential (Figure 6).

It is possible that activation alone of mTORC1 by BCAA treatment led to decreased Rictor protein expression, which was similar to Rictor knockdown, thus inhibiting mTORC2 and activating mTORC1. These findings suggest that mTORC2 may be a true cancer therapeutic target, especially in carcinogenesis related to CSC and the activation of mTORC1 alone leading to decreases in EpCAM and c-myc expression. Thus, we recommend that new cancer therapy strategies aim to inhibit mTORC2 and activate mTORC1 concurrently.

## Supporting Information

Figure S1 **The representative image of EpCAM positive cell which was stained as a method described in Materials & Methods.**
(TIF)

Figure S2 **The percentage of Annexin V-positive cells (**A**) and relative viable cell number (**B**) by array scan in Huh7 cells cultured in DMEM containing 10% FBS with or without 4 mM BCAA in the presence (1 or 2 µg/mL) or absence of 5-FU for 72 h by using target activation protocol of array scan.** Student *t*-test, *p < 0.05, **p < 0.01, n = 7, mean ± SE.
(TIF)

Figure S3 **Tumor volume in the HAK-1B xenograft mouse model on the 14th day after administration of BCAA and 5-FU injection.** Tukey’s test: *p < 0.05, **p < 0.01 vs. control, $$p < 0.01 vs. BCAA, n = 6, mean ± SE.
(TIF)

Figure S4 **The detection of total-p-70S6 kinase in the presence of DMEM, BCAA treatment, pretreatment with rapamycin (**A**) and BCAA treatment, or LC stimulation (**B**) for 72 h in Huh7.**
(TIF)
