# Supplementary figures and images for: Branched Chain Amino Acid Suppresses Hepatocellular Cancer Stem Cells through the Activation of Mammalian Target of Rapamycin
Source: PLoS One. 2013 Nov 27;8(11):e82346. doi: 10.1371/journal.pone.0082346 (PMC3842306; doi:10.1371/journal.pone.0082346)

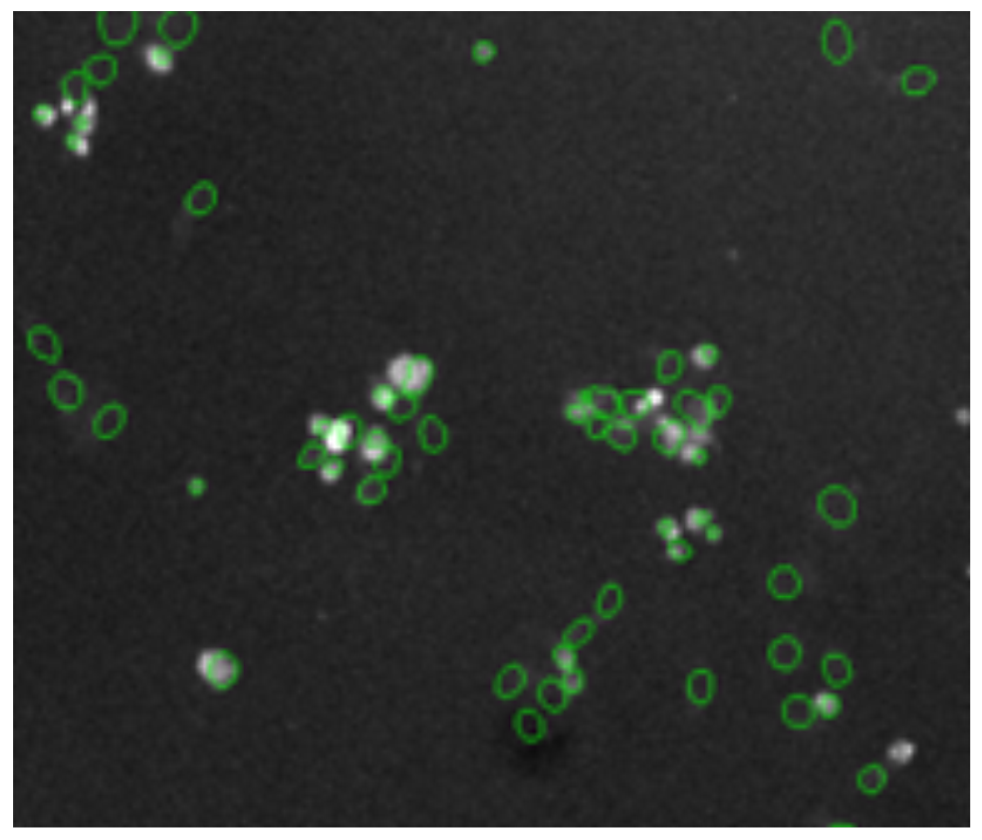

Supplement: Figure S1 — The representative image of EpCAM positive cell which was stained as a method described in Materials & Methods. (TIF) [file pone.0082346.s001.tif]

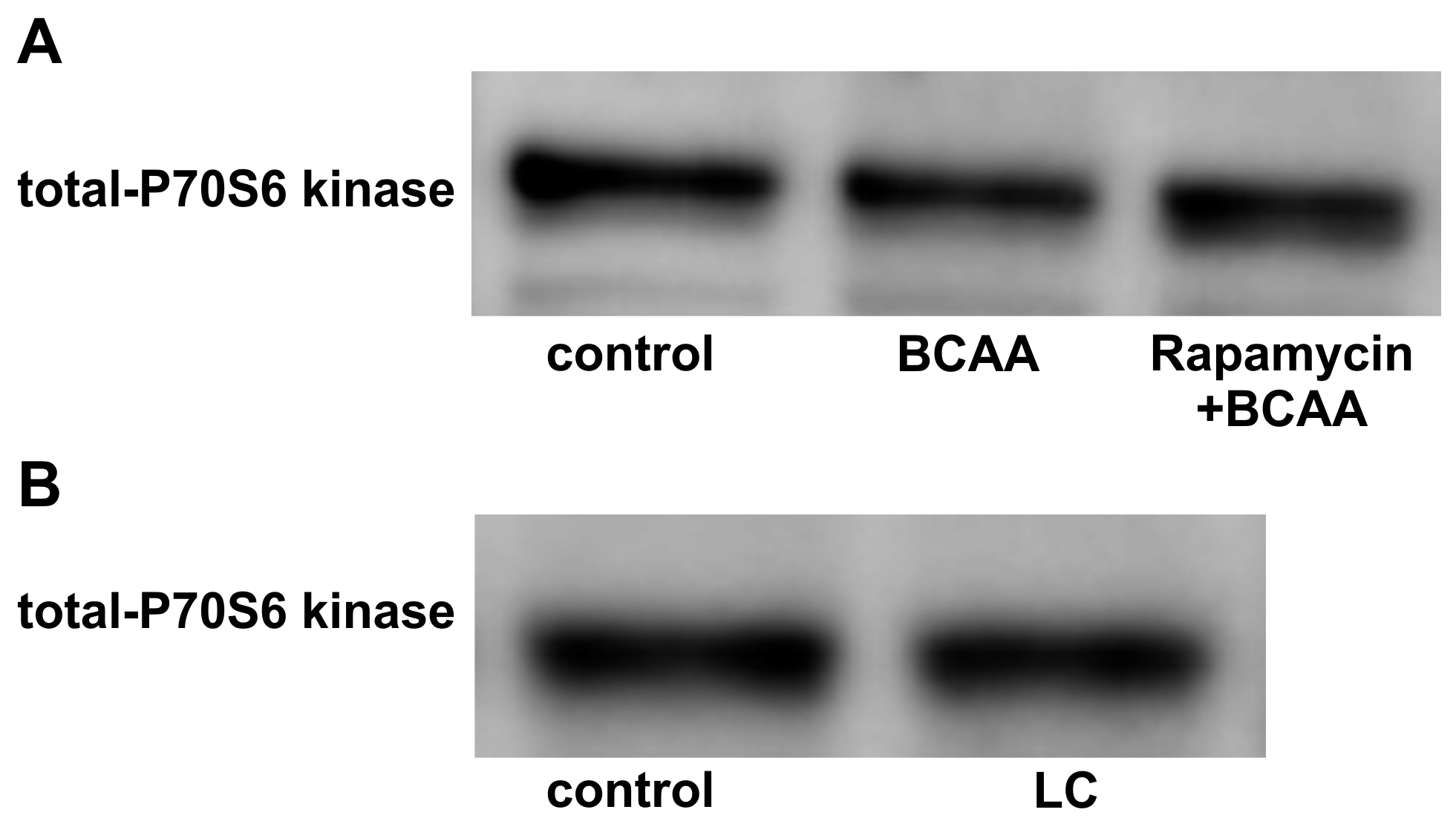

Supplement: Figure S4 — The detection of total-p-70S6 kinase in the presence of DMEM, BCAA treatment, pretreatment with rapamycin (A) and BCAA treatment, or LC stimulation (B) for 72 h in Huh7. (TIF) [file pone.0082346.s004.tif]
